# Computational meta'omics for microbial community studies

**DOI:** 10.1038/msb.2013.22

**Published:** 2013-05-14

**Authors:** Nicola Segata, Daniela Boernigen, Timothy L Tickle, Xochitl C Morgan, Wendy S Garrett, Curtis Huttenhower

**Affiliations:** 1 Biostatistics Department, Harvard School of Public Health, Boston, MA, USA; 2 The Broad Institute of MIT and Harvard, Cambridge, MA, USA; 3 Department of Immunology and Infectious Diseases, Harvard School of Public Health, Boston, MA, USA; 4 Department of Medicine, Harvard Medical School, Boston, MA, USA; 5 Department of Medical Oncology, Dana-Farber Cancer Institute, Boston, MA, USA; 6 Present address: Centre for Integrative Biology, University of Trento, Trento, Italy

**Keywords:** meta'omics, microbial communities, microbiome computational models

## Abstract

Complex microbial communities are an integral part of the Earth's ecosystem and of our bodies in health and disease. In the last two decades, culture-independent approaches have provided new insights into their structure and function, with the exponentially decreasing cost of high-throughput sequencing resulting in broadly available tools for microbial surveys. However, the field remains far from reaching a technological plateau, as both computational techniques and nucleotide sequencing platforms for microbial genomic and transcriptional content continue to improve. Current microbiome analyses are thus starting to adopt multiple and complementary meta'omic approaches, leading to unprecedented opportunities to comprehensively and accurately characterize microbial communities and their interactions with their environments and hosts. This diversity of available assays, analysis methods, and public data is in turn beginning to enable microbiome-based predictive and modeling tools. We thus review here the technological and computational meta'omics approaches that are already available, those that are under active development, their success in biological discovery, and several outstanding challenges.

## Introduction

Microbes and their biochemical activities are an essential component of virtually all ecosystems on earth, shaping environments ranging from deep marine sites to our own body. For example, marine microbial communities are responsible for half of the oxygen produced on our planet (Rocap et al, 2003), and the complex human microbiome complements us with over 100 times more genes than those in our own genome (Qin et al, 2010; The Human Microbiome Project Consortium, 2012b). Host-associated microbes and their biochemical activity have been further linked to healthy and dysbiotic phenotypes, including obesity (Backhed et al, 2004; Turnbaugh et al, 2009a; Kau et al, 2011), Crohn's disease (Manichanh et al, 2006; Morgan et al, 2012), and type 2 diabetes (Qin et al, 2012). Such communities almost always comprise complex mixtures of bacteria, viruses, archaea, and micro-eukaryotes, all of which will be referred to here in combination as microbes.

**Figure 1:**
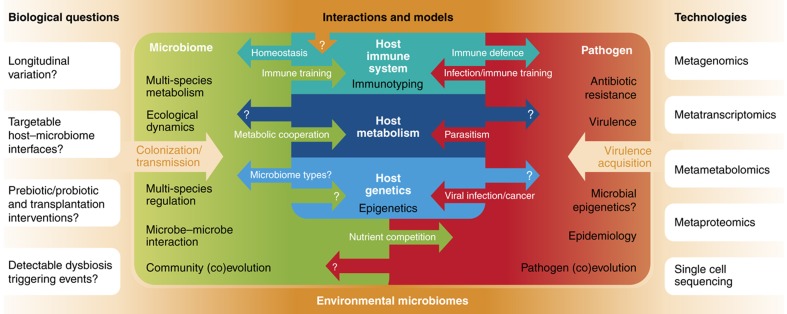
Open biological questions in microbial community biology, and emerging technologies and models for their exploration. Microbial communities are complex biological entities interacting with the environment, host organisms, and transient microbes. Predictive models for most of the interactions within these ecosystems are currently rare, but several studies have begun to provide key insights.

Although the ubiquity and complexity of microbial communities have been well studied for decades, advances in high-throughput sequencing have provided new tools that supplement culture-based approaches both in their molecular detail and in their accessibility to a broad scientific community. The first culture-independent approaches were based on low-throughput sequencing of the bacterial 16S ribosomal rRNA gene (Schmidt et al, 1991; Tringe and Hugenholtz, 2008), and the popularity and effectiveness of 16S-based surveys grew dramatically with increased throughput of sequencing methods. More recently, genome-wide sequencing approaches, such as metagenomics and metatranscriptomics, have further expanded the experimental tools available for studying the microbiome. Such ‘meta'omic' approaches expose the genes, transcripts, and eventually proteins and metabolites from thousands of microbes to analysis of biochemical function and systems-level microbial interactions (Figure 1).

Metagenomic, metatranscriptomic, and other whole-community functional assays provide new ways to study complex ecosystems involving host organisms, biogeochemical environments, pathogens, biochemistry and metabolism, and the interactions among them (Figure 1). Interaction modeling is particularly relevant for human health, and current host–microbe–microbiome systems most often rely on mouse models of the interplay of commensal microbes, pathogens, and hosts. Examples include the ability of the commensal microbiome to eradicate *Citrobacter rodentium* infections (to which germ-free mice are susceptible (Kamada et al, 2012)) and the development of inflammatory colitis and colorectal cancer (Garrett et al, 2010). Pathogen interactions are also well documented with respect to host metabolism and invasion mechanisms (Giannakis et al, 2008; Croxen and Finlay, 2009; Bidle and Vardi, 2011). Findings on host–microbiome interactions with the immune system likewise include concrete host-based mechanisms by which homeostasis is maintained (Ivanov et al, 2009; Hooper et al, 2012) and by which disease-associated dysbiosis develops (Turnbaugh et al, 2010; Kau et al, 2011; Morgan et al, 2012). Conversely, the mechanisms of action by which whole-microbial communities are linked to complex disease, such as carcinogenesis (Kostic et al, 2012) or metabolic phenotypes (Li et al, 2008), are still preliminary and without clear causal directionality. This is also true of the host–microbiome epidemiology, such as initial colonization early in life (Dominguez-Bello et al, 2010; Koenig et al, 2011; Yatsunenko et al, 2012) and the acquisition of virulence and/or drug resistance (Chen and Novick, 2009). In particular, for these emerging areas integrative meta'omic approaches and advanced computational tools are key for a system-level understanding of relevant biomedical and environmental processes, and here we describe current techniques, recent advances, and outstanding challenges.

### Meta'omic sequencing for microbiome studies

A meta'omic study typically aims to identify a panel of microbial organisms, genes, variants, pathways, or metabolic functions characterizing the microbial community populating an uncultured sample. Metagenomics as a term can refer loosely to the field as a whole and to the specific sequencing of whole-community DNA, and it is naturally complemented by metatranscriptomics (cDNA sequencing) and functional technologies, such as metaproteomics and community metabolomics (Wilmes and Bond, 2006; Turnbaugh and Gordon, 2008; Gilbert and Hughes, 2011). Metagenomic and metatranscriptomic approaches in particular assess the genomic composition and diversity within and across microbial communities by means of culture-independent sequencing technologies, including targeted rRNA gene sequencing (16S in bacteria, 18S in eukaryotes, and internal transcribed spacer, typically in fungi (Dollive et al, 2012)) and whole-metagenome shotgun (WMS) sequencing.

WMS sequencing is based on extracting DNA or RNA from the community in its entirety, followed by library construction and short-read sequencing of the entire mixture of genomes or transcripts. The resulting millions of short random DNA/cDNA fragments can then be assembled (often only partially) or used individually as markers for specific organisms and metabolic functions. Compared with rRNA amplicon sequencing, shotgun meta'omics typically provides insight into functionality of microbes and their biological processes, including horizontal gene transfer, sequence variants and evolutionary variability, and genome plasticity. It allows organisms to be identified with increased taxonomic resolution (Tyson et al, 2004; Qin et al, 2010), as the whole genomes of organisms in the community are available for characterization rather than the more limited single 16S/18S molecular clock. The 16S sequencing, of course, remains a more efficient approach to assess the overall phylogeny and diversity of a community, especially when the assayed environment contains a large fraction of uncharacterized microbes. The benefits of WMS sequencing come at the expense of greater cost per sample, although this continues to decrease every year, and of more complex bioinformatic analytical processes (Table I).

The Illumina platform is currently preferred for meta'omic sequencing, and is also supplanting the Roche 454 platform widely used in microbial community analysis for rRNA gene surveys (Bartram et al, 2011; Caporaso et al, 2012). Emerging platforms that have not yet become widely used for microbial community studies, such as Ion Torrent and PacBio, are not discussed in this review. Illumina technologies now produce shorter reads than most alternatives, typically 100 bases for HiSeq and 150 for MiSeq (Qin et al, 2010; Mason et al, 2012; The Human Microbiome Project Consortium, 2012a). These can be contrasted to Roche 454 sequencing technology's ∼500 nt–1 knt reads, which come at the cost of lower throughput and much higher cost per base and read. In both cases, the associated chemistries change rapidly, and short read lengths only infrequently influence meta'omic analyses for assembly-free and marker-based profiling. A recent study (Luo et al, 2012) provides a thorough comparison of Illumina versus Roche 454 for metagenomics by sequencing the same community DNA sample with each platform. The authors found that both platforms agreed on over 90% of the assembled contigs and 89% of the unassembled reads, as well as on the estimated gene and genome abundance in the sample. Illumina sequence quality was additionally less affected than that of 454 when comparing frameshift errors in technical replicates. They concluded that both technologies are reliable for quantitatively assessing diversity within natural communities, although the Illumina platform provides higher coverage and lower cost than Roche 454.

### Assembly-based microbial community analysis

Metagenomic sequencing, if performed at a sufficiently high coverage, can in some cases allow reconstruction of complete genomes of organisms in a community (Culley et al, 2006; Hess et al, 2011; Narasingarao et al, 2012). In practice, the high complexity of many typical communities leads to chimeras and unresolvable ambiguities in scaffold reconstruction due to conserved DNA regions, organismal variability, and horizontal gene transfer (Pignatelli and Moya, 2011; Mende et al, 2012). Despite these theoretical limitations, even early work with single-genome assembly approaches like SOAPdenovo has resulted in good, although sometimes fragmentary, reconstruction of highly abundant microbes from metagenomics (Qin et al, 2010; The Human Microbiome Project Consortium, 2012a).

However, recent years have seen an explosion of metagenome-specific assemblers, which use strategies to tease apart sequencing artifacts from true biological ambiguity within communities. Only a subset of these includes MetaVelvet (Namiki et al, 2012), khmer (Pell et al, 2012), metAMOS (Treangen et al, 2011b), Meta-IDBA (Peng et al, 2011), and MetaORFA (Ye and Tang, 2009). Metagenomic assemblers generally adapt graph-based reconstruction approaches to account for variability in genome copy number and an increase in unresolvable ambiguities caused by sequences conserved in multiple genomes. These are thus powerful and promising tools to study low-to-medium complexity microbiomes, or abundant organisms in novel complex communities, without relying on previously sequenced isolates. One such successful report isolated a marine archaeal genome and its symbionts from oceanic oxygen minimum zones (Narasingarao et al, 2012).

**Figure 2:**
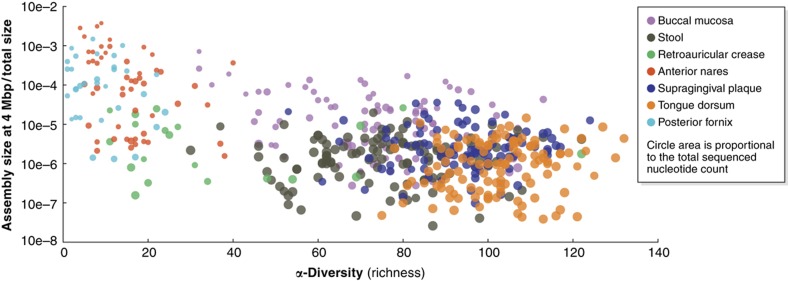
Community diversity and metagenome depth interact to influence assembly quality. Five hundred and twenty-two metagenomic assemblies from the Human Microbiome Project (HMP) are shown here to demonstrate the complex interaction of underlying microbial α-diversity (x axis, diversity within a sample measured as species richness) and assembly quality (y axis). The latter was measured as the size of the smallest contig such that the cumulative length of longer contigs exceeds 4 Mbp, normalized by the total sequenced microbial nucleotide count (The Human Microbiome Project Consortium, 2012a). Communities from each of the seven available body sites are highlighted in different colors, with each point's area proportional to the total input nucleotides for assembly. Microbial composition, metagenome depth, and assembly approach (not shown) all interact to greatly influence the resulting assembly quality.

Whole-genome assembly from metagenomes is impossible in most cases, and such assemblers instead aim to provide the largest reliable and useful contigs achievable from their input sequence reads (Figure 2). Although having access to the synteny of microbial genes within communities is invaluable to unravel their complete genomic features, it is rare that the quality of whole genomes isolated from metagenomes approaches that of cultured isolates, and particular care should be devoted to avoiding (partially) chimeric genomes. For relatively well-characterized environments, however, accurate microbial community profiles can be obtained even for complex communities by exploiting the ever-increasing collection of sequenced microbes.

### Community profiling using prior genomic knowledge

**Figure 3:**
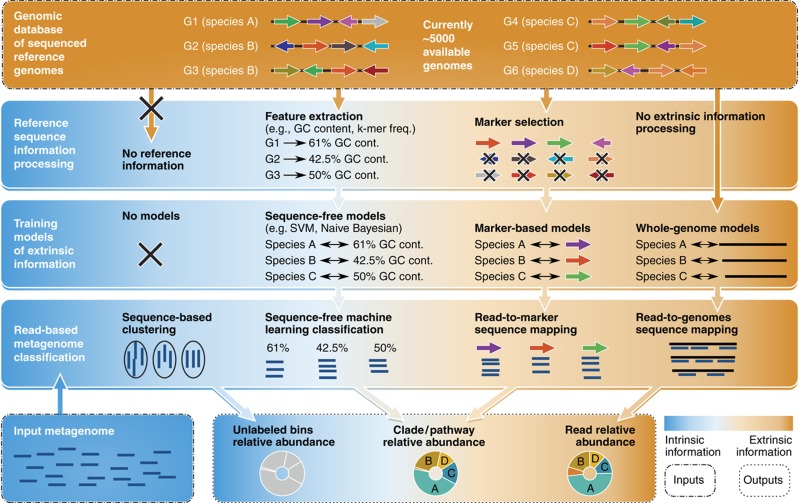
Intrinsic versus extrinsic metagenomic analysis can minimally, partially, or completely rely on prior knowledge from sequenced reference genomes. Methods that do not rely on any reference sequence information typically perform a sequence-based clustering of meta'omic reads, resulting in unlabeled clusters of sequences that can later be assigned to taxonomic or functional classes (analogous to Operational Taxonomic Unit clustering for 16S sequences). Available genomes can alternatively be used more extensively as references for short-read mapping, typically incurring an expense of high computational cost and possible ambiguous assignments for reads from nonunique regions. Intermediate approaches typically rely on a combination of pre-processing extrinsic reference genome information (e.g., to train a composition-based classifier) and intrinsic information (e.g., reads' nucleotide composition) to improve the discrimination power and focus the subsequent mapping operation to the most discriminative sequence-based markers.

Identifying the organisms populating a microbial community and their proportions (relative abundances) is the typical primary objective of amplicon sequencing investigations (e.g., 16S rRNA gene surveys). Metagenomic shotgun sequencing can provide comparable information, often at better resolution, either by *de novo* binning of microbial sequences (using intrinsic sequence properties) or by identifying them using information from sequenced microbial genome databases (extrinsic information, Figure 3). Similarly, these profiling tasks can be performed for metagenomes by attempting to classify every individual read, by assembly and binning of entire contigs, or by profiling summary information (e.g., k-mer profiles) for the entire community in aggregate.

All of these approaches rely in some way on reference genome catalogs. Although those sequenced for microbial organisms are biased towards model organisms and pathogens, large-scale efforts like the Human Microbiome Project (Nelson et al, 2010; Fodor et al, 2012)) and the Genomic Encyclopedia of Bacteria and Archaea (Wu et al, 2009) are systematically filling the gaps in the sequenced portion of the phylogeny. Such efforts take advantage of a variety of innovative isolation approaches, including culture-independent techniques, host monocolonization (Sczesnak et al, 2011), single-cell sequencing (Pamp et al, 2012), and, modulo the limitations above, metagenomic assembly. Consequently, a reference set of microbial genomes on the order of 5000 finished or high-quality sequences are now available (Markowitz et al, 2012a), describing more than 2000 species, and these numbers are quickly increasing. Comparing metagenomes with this compendium of reference genomes thus provides a variety of ways to ease the task of community profiling by providing additional taxonomic and phylogenetic information.

Intrinsic binning approaches for community profiling (Figure 3, leftmost panels) typically train a taxonomic (or phylogenetic) classifier from reference genomes and then use this sequence-free classifier to bin new meta'omic reads. These have included statistical approaches, such as Support Vector Machines with structured output (PhyloPythiaS (Patil et al, 2012)), interpolated Markov models (Phymm (Brady and Salzberg, 2011)), naive Bayesian classifiers (Rosen et al, 2011)), and Self Organizing Maps (TaxSOM (Weber et al, 2010)), or integration of intrinsic and homology-based extrinsic assignments (PhymmBL (Brady and Salzberg, 2011), RITA (Parks et al, 2011)). An even more reference-independent approach is possible by using only composition-based clustering (e.g., TETRA (Teeling et al, 2004)), which can then be paired with further downstream analysis. For environments with insufficient genomic prior information, sequence-based (intrinsic) or hybrid approaches perform substantially better than the homology-based ones, but they typically require very long running times due to the large sizes of both metagenomic data and the reference sequence repositories.

Extrinsic or homology-based classification (Figure 3, rightmost panels) instead relies directly on comparisons of metagenomic sequences with reference sequences in order to identify taxonomic or phylogenetic origin. Several alternatives to whole-genome searches have been developed, relying on the extraction of only the most informative features from reference genomes to reduce the complexity of mapping operations. Extracting only the 16S rRNA genes for profiling from a metagenome is an extreme example of this approach, and expanding such universal markers to include additional highly conserved genes further improves generalizability and phylogenetic resolution. AMPHORA (Wu and Scott, 2012) adopts 31 such markers (mainly ribosomal proteins as identified by Ciccarelli et al (2006), whereas MetaPhyler (Liu et al, 2011) and AMPHORA2 (Wu and Scott, 2012) complement these bacterial markers with additional archaeal genes. Even different strains within the same microbial species can be discriminated by supplementing this approach with more diverse gene sets; MetaPhlAn (Segata et al, 2012) adopts such a strategy by pre-identifying unique clade-specific marker genes as species-specific name tags. This provides hundreds of markers for most species, increasing robustness and permitting more precise organismal abundance estimation. By using such targeted data, all of these marker-based approaches can achieve computational run times orders of magnitude faster than using full genomes as mapping targets for metagenomes.

The most extrinsic methods for binning and community profiling instead use whole-genome searches of metagenomic sequences against the entire catalog of reference genomes. This can be performed with varying levels of sensitivity by using homology search (e.g., BLASTN (Altschul et al, 1997)) or mapping (e.g., BowTie2 (Langmead and Salzberg, 2012), or BWA (Li and Durbin, 2010)). However, such results can be highly ambiguous and difficult to interpret because of evolutionarily conserved or horizontally transferred sequences. These are taxonomically aspecific and, when relied on for profiling, cause inconsistencies such as long tails of false-positive organisms. Computational modeling of this mapping permits to correct most profiling issues and provide accurate taxonomic characterization of the metagenomic reads; phylogenetic approaches typically rely on assigning reads to the lowest common ancestor of the taxa with hits (MEGAN (Huson et al, 2007)) or other parsimonious evolutionary principles (PaPaRa (Berger and Stamatakis, 2011)). Of course, such complete genome lookups provide further utility beyond organismal profiling, such as information on individual microbial sequence variants and on the gene and pathway repertoires of a community.

### Gene function annotation and metabolic reconstruction

Microbial communities can be seen not only as groups of individual microbes, but also as collections of biochemical functions affecting and responding to an environment or host organism. Metagenomics can thus also identify the genes and pathways carried by a microbial community, and metatranscriptomics can profile their expressed function. Just as several alternatives for microbial profiling are described above, two broad classes of functional community profiling depend either on genes identified within longer assembled contigs or on assembly-free read-based approaches. Assembly-based methods are sensitive to the challenges outlined above, such as a bias towards higher-abundance community members or sequences that are easy to assemble. Assembly-free functional inference, by mapping sequences to annotated reference genomes or functional databases, can be more sensitive (including a greater proportion of reads or microbes) but less specific in its functional identifications.

Assembly-based metagenome annotation can be performed by adapting pipelines for annotation of single microbial genomes. Metagenomic contigs are thus scanned for identifying protein-coding genes (CDSs), as well as CRISPR repeats, noncoding RNAs, and tRNA. Functional characterization can then be performed assigning full CDSs (rather than single-sequencing reads) to functional categories by means of orthology relations with sequences in well-characterized functional databases, such as NCBI nr (Pruitt et al, 2012), the KEGG Orthology (Kanehisa and Goto, 2000), and COGs (Tatusov et al, 1997), or by identifying specific PFAM (Punta et al, 2012) or SMART (Schultz et al, 1998) peptide domains within CDSs. Broader biological functions are then built on these low-level functional annotations (Mitra et al, 2011) using hierarchical ontologies that group functionally related proteins as in KEGG (Kanehisa and Goto, 2000), MetaCyc (Caspi et al, 2012), and SEED (Overbeek et al, 2005). Integrated pipeline are also available (Meyer et al, 2008; Markowitz et al, 2012b) to automate these bioinformatic tasks.

Functional profiling using reference information can be based either on reference genome read mapping (at the nucleotide level) or on translated protein database searches. For the former, reads mapped to reference genomes as discussed above can then be sent through an additional second mapping from loci to annotated functions. For the latter, functional databases of diverse protein families as listed above can be leveraged to identify function by translated homology search. Like reference genomes, these databases are generally also enriched for functional information from model organisms and pathogens. Thus, the greater the enrichment of such organisms in a community, the more characterized functional annotations are likely to be retrieved. Examples of pipelines, including functional annotation by translated mapping, include MG-RAST (Meyer et al, 2008), MEGAN (Huson et al, 2007), and HUMAnN (Abubucker et al, 2012). Each of these methods typically includes some combination of additional quality control and interference steps subsequent to homology search, such as selection of pathways by maximum parsimony, taxonomic limitation, or statistical smoothing (Abubucker et al, 2012).

It is important to note that whole-community functional profiling is not yet a mature area, and neither gene annotations within reference genomes nor those in protein databases are well tuned to whole-community metabolism. For example, MetaCyc (Caspi et al, 2012) and SEED (Overbeek et al, 2005) both have ongoing efforts to develop microbiome-specific functional annotations, and gene family catalogs, such as eggNOG (Powell et al, 2012), are intended to eventually better represent uncultured communities. Leveraging these functional annotations after they are profiled will likewise require further improvements, both in more nuanced function identifications (e.g., ‘glycosyltransferase' as opposed to ‘carbohydrate processing') and in the identification of gene products' localization upon translation (e.g., secretion or compartmentalization). Finally, algorithms for nucleotide search (Li and Durbin, 2010; Langmead and Salzberg, 2012) have outpaced those needed for translated mapping (i.e., BLASTX (Altschul et al, 1997) and USEARCH (Edgar, 2010)), and bioinformatic advances will further improve the translated search.

### Microbial ecosystem interaction and association networks

Knowledge of the microbes and gene products within communities is an important step toward understanding their ecology, environmental responses, and interorganismal interactions (DeLong and Pace, 2001). Microbial communities are shaped by the same highly diverse coexistence patterns that occur in other ecologies. These include interspecies and intercellular relationships of a range of symbiotic interaction types: win–win (mutualism), lose–lose (competition), win–lose (parasitism, predation), win–zero (commensalism), or zero–lose (amensalism). These are based on processes such as microbial organisms exchanging or competing for nutrients, and they have long been studied by nonsequence-based approaches (Konopka, 2009). Detecting such microbial interactions in communities and identifying their mechanisms is a daunting bioinformatic challenge. Even the best meta'omic profiles contain substantial measurement error and, more importantly, represent compositional data that cause extreme biases when analyzed using most correlation or co-occurrence measures (Lovell et al, 2010; Pawlowsky-Glahn and Buccianti, 2011). To date, several similarity measures have been used for determining co-occurring or co-excluding microbial relationships, including Pearson's or Spearman's correlation (Qin et al, 2010), hypergeometric overlap tests for species presence/absence data (Chaffron et al, 2010), and mutual information. The behavior of these measures in sparse, compositional microbial abundance data is unlikely to be appropriate in most biological settings.

To assess meta'omic profiles more accurately, recent association approaches have been developed specifically for intermicrobe co-occurrence and co-exclusion detection in microbial communities. For example, Faust et al (2012) combined similarity measures with a composition-sensitive, nonparametric statistical test to predict microbial relationships within and between body sites in the human microbiome. SparCC (Friedman and Alm, 2012) is another novel approach that quantifies the composition-free component of Pearson's correlation values from microbial relative abundances. Lozupone et al (2012) used the Bray–Curtis distance, only partially sensitive to compositions, as a co-occurrence measure for network inference to identify genomic and metabolic features in human gut symbionts. Association of microbial variation and covariation with environmental parameters (e.g., host biogeography, temperature, pH, etc. (Raes et al, 2011)) is a distinct task for which employing the correct statistical methodology can be challenging. It remains an area of active research, with current options including categorical nonparametric biomarker discovery (White et al, 2009; Parks and Beiko, 2010; Segata et al, 2011) and appropriately transformed regression models (Chen et al, 2012).

All of these current approaches, however, identify only the descriptive covariation of multiple microbes; they characterize neither the mechanisms of nor the regulatory ramifications of such variation. There is thus a pressing need for multi-organism metabolic models to explain such interactions (Klitgord and Segrè, 2010; Bucci et al., 2012) and for a systems-level understanding of their effect on microbial signaling and growth (Zengler and Palsson, 2012). Both will rely on better gene function annotations as mentioned above, particularly on improved catalogs of intermicrobial small molecule and peptide signaling mechanisms. Careful experimental validation, including both *in vitro* culture and *in vivo* dynamics, will be needed to ensure the correctness of these challenging models; some studies of the latter with respect to natural long-term dynamics (McCarren et al, 2010; Gajer et al, 2012; Zhao et al, 2012) and short-term perturbations (Dethlefsen and Relman, 2011; Ubeda et al, 2013) have already begun. Such interactions must, of course, also account for the host in host-associated communities, where host–microbe interactions can comprise both direct protein interactions and metabolic (i.e., nutrient) interdependencies (Kinross et al, 2011). In particular, with respect to adaptive and innate immunity, examples such as segmented filamentous bacteria emphasize the importance of complex interaction of microbes with host development. The presence of this organism is sufficient to drive drastic changes in gut physiology and T-cell differentiation in mice (Ivanov et al, 2009; Atarashi et al, 2011), but neither its nor other microbes' roles in human cell signaling or development have yet been well explored.

### Unraveling community expression patterns with metatranscriptomics

Most current meta'omic tools and studies focus on metagenomic DNA sequencing, but metatranscriptomics is becoming increasingly practical as a window into the regulation and dynamics of microbial community transcription. Similar to metagenomics, studies of microbial community gene expression emerged from marine research (Frias-Lopez et al, 2008; Shi et al, 2009; Gilbert and Hughes, 2011). These revealed not only gene- and taxon-specific expression patterns but also gene categories undetected in previous DNA-based surveys (Frias-Lopez et al, 2008) and nonprotein-coding small RNAs in naturally occurring microbial communities (Shi et al, 2009). Few studies have so far analyzed microbial gene expression in host-associated communities, as this can present greater technical challenges in isolating a sufficient quantity of microbial (rather than host) transcript. Recent investigations have included the murine intestine (Turnbaugh et al, 2009b), the healthy human gut (Gosalbes et al, 2011; McNulty et al, 2011), the microbiota of monozygotic twins (Turnbaugh et al, 2010), and the airways of cystic fibrosis patients (Lim et al, 2012). These studies profiled whole-community cDNAs and compared them with metagenomic DNA, a critical step in metatranscriptomic interpretation. Unlike single-organism genomes, both the transcript copy number and genomic copy number can easily change in microbial communities, rendering this normalization an important computational step (Shi et al, 2011).

The major challenge faced in metatranscriptomics is the isolation of microbial mRNA, which usually makes up only a small percentage of total microbial RNA and an even smaller proportion of total RNA if host nucleotides are present. Eukaryotic genes and genomes are sufficiently large as to rapidly swamp smaller microbial transcripts, and even in nonhost-associated communities over 90% of microbial transcripts are typically ribosomal rRNA. The difficulty of isolating prokaryotic mRNA is further compounded by its lack of the 3′-end poly (A) tail that marks eukaryotic mRNA (Gosalbes et al, 2011). High-quality commercial rRNA depletion kits are available (such as Ribo-Zero, RiboMinus, and QIAGEN GeneRead), but even removal of the majority of such sequences can leave substantial ‘wasted' sequencing reads that must be computationally depleted *post hoc*. Likewise, although physical depletion of host sequences is an area of active technology development, computational postprocessing (e.g., by mapping to host genomes) remains the most practical current approach in whole-community analysis.

### Meta'omics with single-cell resolution

Single-cell sequencing provides an alternative approach to accessing novel information about uncultured microbes (Lasken, 2012). Although it currently incurs high costs per sample and per depth of sequencing relative to metagenomics, it can cleanly circumvent both host sequence contamination and the difficulty of metagenomic assembly. Single-cell isolation can sequence low-abundance organisms at higher resolution than metagenomic approaches as well, with a corresponding tradeoff in its breath of profiling for more diverse communities. This provides high resolution for individual organisms as well, allowing a subset of the exact strains present in a sample to be readily identified. This provides a starting point for tasks that can be challenging in the WMS data, such as detecting strain variability across time or subjects. Goodman et al (2011) showed that the human fecal microbiota consists largely of taxa and predicted functions that are represented in its readily cultured members by combining high-throughput anaerobic culturing techniques with gnotobiotic animal husbandry and metagenomics. Their study also revealed that thousands of isolates from a single donor can be clonally archived and taxonomically mapped in multi-well format to create personalized microbiota collections.

Current single-cell approaches first isolate single microbial cells by sorting them, lyse them separately, amplify and label them separately, and sequence the resulting pool. The subsequent analysis of single-cell sequence data thus relies much more heavily than do meta'omics on assembly, but fortunately in a less-challenging setting. IDBA-UD (Peng et al, 2012) and SmashCell (Harrington et al, 2010) provide some of the first software environments for assembling and annotating such data, and commercial technologies such as RainDance (Lexington, MA) and microfluidics platforms are emerging to isolate single microbial cells with high quality. Recent applications in microbial communities have ranged from environmental samples like seawater (Woyke et al, 2009; Mason et al, 2012) and soil (Kvist et al, 2007) to hosts such as insects (Woyke et al, 2010), mice (Pamp et al, 2012), and humans (Marcy et al, 2007). Recently, elegant combinations of both single-cell genomics and metagenomics have begun to emerge, e.g., in the sequencing of a novel, low-salinity ammonia-oxidizing archaeon from an enrichment culture (Blainey et al, 2011). Such a combinatorial approach may continue to prove very useful, as the single-cell perspective on novel organism-specific sequences tends to complement whole-metagenome and metatranscriptome overviews of diverse communities.

### Models of microbiome evolution and coevolution

Meta'omics provides an important tool for studying evolution within microbial communities, which can occur on two very different time scales. Over the course of days, weeks, or the years of a host's lifetime, microbial genome plasticity allows remarkably rapid acquisitions of novel mutations and laterally transferred genes. Over the course of millennia, however, the overall structure of host-associated communities, their phylogenetic composition, and their microbial pan-genomes can evolve more slowly in tandem with their hosts' physiology and immune systems (Lefébure and Stanhope, 2007).

Our current understanding of short-term microbial coevolution arises mainly from the study of human pathogens, which are subject to the enormous evolutionary pressures of immune evasion and treatments such as antibiotics. Such pressures affect the entire microbial communities, of course, and not only single pathogens but, before meta'omic sequencing, this was also difficult to quantify. Both single-nucleotide polymorphisms and lateral gene transfer/recombination have recently been shown to arise *in vivo* (Croucher et al, 2011; Lieberman et al, 2011), with evidence suggesting the latter is particularly frequent among microbes that stably inhabit shared communities (McDaniel et al, 2010; Smillie et al, 2011). The distribution of antibiotic resistance throughout a community is of particular interest in this respect as a public health concern, as convergent evolution of resistance polymorphisms (Croucher et al, 2011) and transient lateral transfer from less proximal environments (Hehemann et al, 2010; Forsberg et al, 2012) have both been observed for this phenotype and for other evolutionary pressures.

Over macro-evolutionary time scales, host-associated microbiomes in particular have developed exquisite symbioses with both plant and animal hosts. For example, some of the earliest evidence of microbial symbiosis focused on the role of rhizobia in legume root development and nitrogen acquisition (Hakoyama et al, 2009). In vertebrates, the Hawaiian bobtail squid has emerged as a remarkable system demonstrating selective microbial adaptation. The light organ of this squid is sterile at hatching, but is subsequently selective for a finely tuned *Vibrio* bacterial population that provides it with bioluminescence that enables the squid to avoid predation (McFall-Ngai, 2008; McFall-Ngai et al, 2011). Potential genomic impacts of long-term host–symbiont relationships are described in symbiont–insect codiversification, many of which have been approximated of upwards of 180 millions years old (Moran et al, 1993). Conversely, intracellular microbial symbionts can exhibit dramatically, and often unusually, reduced genomes owing to close integration with their hosts (Moran et al, 2008). Less-understood mutualism occurs in the human gut, which is one example of a wide range of microbiome configurations that have evolved to leverage diverse mammalian guts and diets (Ley et al, 2008; Muegge et al, 2011). Characterizing the coevolution of quickly evolving complex microbial communities with relatively slowly evolving eukaryotic hosts remains a challenging and largely unexplored field.

### Predictive bioinformatic models and model microbial communities

One of the ultimate goals of microbial community systems biology is to develop predictive models of the whole-community response to changing stimuli, be it their temperature or pH in the environment, or dietary components in a host gut. Such models may be mechanistic, relying on joint metabolic networks as discussed above, or a descriptive systems biology of microbial physiological ‘rules' may emerge as a simpler alternative. No unifying approach yet exists, although meta'omic data have provided training input for several first attempts. An artificial neural network-based approach was used to predict ocean-water bacterial community as a function of the marine environment, for which biological validation is challenging (Southward et al, 2005; Larsen et al, 2012). A related methodology, Predicted Relative Metabolomic Turnover, leverages changes in inferred microbial enzyme activity to predict environmental ocean metabolites (Larsen et al, 2011). Joint metabolic predictions have been made to model a set of two- and three-microbe interactions (Klitgord and Segrè, 2010), but in all of these cases biological testing and evaluation has remained a bottleneck. In the absence of extensive functional data for validation, such as metatranscriptomic, metabolic, or proteomic measurements, predictive modeling remains speculative.

Given the complexity of most ‘wild' microbial communities, one of the most promising approaches for such validation has been in the construction of model microbial communities. These have been successful both entirely *in vitro*, by scaling up the *ex vivo* coculture of multiple organisms, and when associated with hosts *in vivo*. Many studies have grown human-derived microbial communities in chemostats (Marsh et al, 1983; McBain and MacFarlane, 2001), with one of the most complex being the Simulator of the Human Intestinal Microbial Ecosystem model, a five-stage multi-chamber chemostat, simulating human digestion (stomach, small intestine, and large intestine) as exposed to foods or pharmaceuticals (Molly et al, 1993). Recent clinical translation of *in vitro* communities has demonstrated success as a treatment for chronic *C. difficile* (Petrof et al, 2013). *In vivo*, the Altered Schaedler Flora (ASF) is a synthetic community transferrable to gnotobiotic mice that has been in use as an experimental system for years (Dewhirst et al, 1999). The eight-microbe ASF and similar models are enjoying a resurgence as a simpler alternative to hundred-organism natural communities in which to mechanistically assess microbe–microbe and host–microbe molecular interactions. An end-to-end demonstration of this concept was carried out in the Gordon lab, using a gnotobiotic mouse model colonized with a custom synthetic microbial community, followed by systematic dietary perturbations to train and then validate predictive models of the community's response (Faith et al, 2011).

## Conclusions and outlook

**Figure 4:**
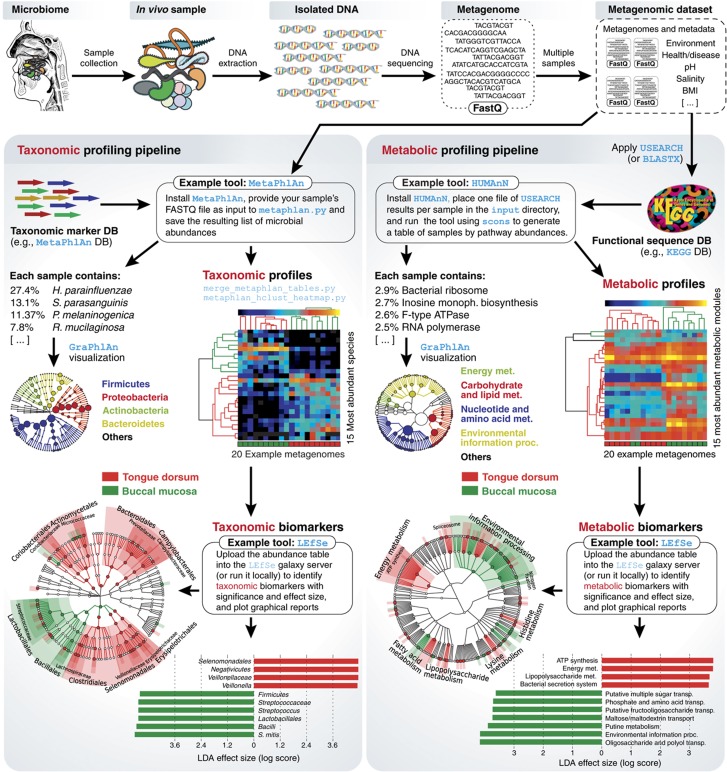
A typical current computational meta'omic pipeline to analyze and contrast microbial communities. After collecting microbiome samples, community DNA or RNA is extracted and sequenced, generating WMS samples (i.e., metagenomes) generally consisting of several million short reads each. This example uses 20 WMS samples from the oral cavity (10 from the buccal mucosa, and 10 from the tongue dorsum (The Human Microbiome Project Consortium, 2012b)). Complementary methods reconstruct the taxonomic characteristics (left) and metabolic potential (right) of the microbial communities. MetaPhlAn (Segata et al, 2012) is one of many alternatives to detect and quantify microbial clades with species-level resolution (see Section 3), whereas HUMAnN (Abubucker et al, 2012) quantitatively characterizes genes, pathways, and metabolic modules from each community (see Section 4). Differentially abundant clades or pathways can then be identified and assessed by tools such as LEfSe (Segata et al, 2011) and represented graphically (e.g., here by GraPhlAn, http://huttenhower.sph.harvard.edu/graphlan). The step-by-step computational pipeline used to produce the analyses reported here is included as a tutorial in Supplementary Information and can also be downloaded from https://bitbucket.org/nsegata/metaphlan/wiki/MetaPhlAn_Pipelines_Tutorial. See Table I for alternative computational approaches to each of these currently common steps in meta'omic analysis.

Although technologies and analyses are constantly improving, WMS sequencing is currently reaching maturity in the sense that validated, standardized experimental and bioinformatic procedures are available to answer typical biological questions of interest (Figure 4 and tutorial in Supplementary Information). These include assessment of the taxonomic and phylogenetic composition of microbial communities at a level of resolution beyond that of individual marker genes, as well as quantification of biomolecular features, including gene families, pathways, metabolism, and functional modules. Statistical methods for biomarker discovery and, in some cases, phenotype prediction can then be performed (Table I). Other meta'omic approaches, such as metatranscriptomics, metaproteomics, and metabolomics, are still under rapid development, with neither experimental nor computational pipelines yet attaining a comparable degree of standardization. These will be crucial to effectively investigate microbial community transcriptional regulation, metabolites dynamics, and protein signaling.

An exciting next step in microbial community systems biology will be the opportunity to integrate and meta-analyze multiple data sets. This is already starting to be the case with large 16S and, gradually, metagenomic data sets defining healthy human microbial baselines (Qin et al, 2010; Yatsunenko et al, 2012; The Human Microbiome Project Consortium, 2012b). Just as with early efforts at microarray and genome-wide association study meta-analysis, systematic differences between diverse projects' platforms and protocols induce strong technical differences between data sets, but these are gradually being overcome (Bittner et al, 2010; Su et al, 2011). However, the integration of complementary data types within the same study, such as joint community metatranscriptomes, metaproteomes (Verberkmoes et al, 2009; Li et al, 2011), and metametabolomes (Jansson et al, 2009), will provide an even richer picture of dynamic microbial systems (Kau et al, 2011). The patterns of tandem host biomolecular activities, or of host or microbial epigenetics (e.g., histone modifications and methylation patterns), remain almost completely unexplored at the whole-community level.

The degree to which microbial community activity and structure is dynamic over time has perhaps been underappreciated, and an additional component necessary for whole-community modeling will be the combination of longitudinal surveys (Koenig et al, 2011; Patil et al, 2011) with systematic perturbation experiments. Early microarray studies involved both time courses in response to chemical stimuli and systematic genetic knockouts in model organisms (Gasch et al, 2000; Hughes et al, 2000). Few such experiments have been pursued in microbial communities, and indeed the concept of a community ‘knock-out' or ‘knock-in' is not yet well explored. Synthetic communities offer a particularly promising avenue for systematically adding or removing organisms, or (in genetically tractable systems) adding or removing single microbial genes. In combination with innovative computational models, meta'omics in such environments and *in vivo* will continue to improve our understanding of microbial community systems biology.

## Supplementary Material

Supplementary Information Step-by-step guide into the computational pipeline of Figure 4

## Figures and Tables

**Table 1:** Current computational methods for meta'omic analysis

Method	Description	Reference
*Assembly*		
Genovo	Generative probabilistic model of reads	(Laserson et al, 2011)
khmer	Probabilistic de Bruijn graphs	(Pell et al, 2012)
Meta-IDBA	De Bruijn graph multiple alignments	(Peng et al, 2011)
metAMOS	A Modular Open-Source Assembler component for metagenomes	(Treangen et al, 2011a)
MetaVelvet	De Brujin graph coverage and connectivity	(Namiki et al, 2012)
MOCAT	Assembly and gene prediction toolkit	(Kultima et al, 2012)
SOAPdenovo	Single-genome assembler commonly tuned for metagenomes	(Li et al, 2010)
MetaORFA	Gene-targeted assembly approach	(Ye and Tang, 2009)
		
*Taxonomic profiling*		
Amphora, Amphora2	Automated pipeline for Phylogenomic Analysis	(Wu and Scott, 2012)
CARMA3	Taxonomic classification of metagenomic shotgun sequences	(Gerlach and Stoye, 2011)
ClaMS	Classifier for Metagenomic Sequences	(Pati et al, 2011)
DiScRIBinATE	Distance Score Ratio for Improved Binning and Taxonomic Estimation	(Ghosh et al, 2010)
INDUS	Composition-based approach for rapid and accurate taxonomic classification of metagenomic sequences	(Mohammed et al, 2011a)
MARTA	Suite of Java-based tools for assigning taxonomic status to DNA sequences	(Horton et al, 2010)
MetaCluster	Binning algorithm for high-throughput sequencing reads	(Wang et al, 2012)
MetaPhlAn	Profiles the composition of microbial communities from metagenomic shotgun sequencing data	(Segata et al, 2012)
MetaPhyler	Taxonomic classifier for metagenomic shotgun reads using phylogenetic marker reference genes	(Liu et al, 2011)
MTR	Taxonomic annotation of short metagenomic reads using clustering at multiple taxonomic ranks	(Gori et al, 2011)
NBC	Naive Bayes Classification tool for taxonomic assignment	(Rosen et al, 2011)
PaPaRa	Aligning short reads to reference alignments and trees	(Berger and Stamatakis, 2011)
PhyloPythia	Accurate phylogenetic classification of variable-length DNA fragments	(Patil et al, 2012)
Phymm, PhymmBL	Classification system designed for metagenomics experiments that assigns taxonomic labels to short DNA reads	(Brady and Salzberg, 2011)
RAIphy	Phylogenetic classification of metagenomics samples using iterative refinement of relative abundance index profiles	(Nalbantoglu et al, 2011)
RITA	Classifying short genomic fragments from novel lineages using composition and homology	(Parks et al, 2011)
SOrt-ITEMS	Sequence orthology-based approach for improved taxonomic estimation of metagenomic sequences	(Monzoorul Haque et al, 2009)
SPHINX	Algorithm for taxonomic binning of metagenomic sequences	(Mohammed et al, 2011b)
TACOA	Taxonomic classification of environmental genomic fragments using a kernelized nearest neighbor approach	(Diaz et al, 2009)
Treephyler	Fast taxonomic profiling of metagenomes	(Schreiber et al, 2010)
		
*Functional profiling*		
HUMAnN	Determines the presence/absence and abundance of microbial pathways in meta'omic data	(Abubucker et al, 2012)
metaSHARK	A web platform for interactive exploration of metabolic networks	(Hyland et al, 2006)
PRMT	Predicted Relative Metabolomic Turnover: determining metabolic turnover from a coastal marine metagenomic dataset	(Larsen et al, 2011)
RAMMCAP	Rapid analysis of Multiple Metagenomes with Clustering and Annotation Pipeline	(Li, 2009)
		
*Interaction networks*		
SparCC	Estimates correlation values from compositional data for network inference	(Friedman and Alm, 2012)
CCREPE	Predicts microbial relationships within and between microbial habitats for network inference	(Faust et al, 2012)
		
*Single-cell sequencing*		
IDBA-UD	Assembler for single-cell or metagenomic sequencing with uneven depths	(Peng et al, 2012)
SmashCell	Software framework for the analysis of single-cell amplified genome sequences	(Harrington et al, 2010)
		
***Simulators***		
GemSIM	Error-model based simulator of next-generation sequencing data	(McElroy et al, 2012)
MetaSim	A sequencing simulator for genomics and metagenomics	(Richter et al, 2008)
		
*Statistical tests*		
Metastats	Statistical analysis software for comparing metagenomic samples	(White et al, 2009)
LefSe	Nonparametric test for biomarker discovery in proportional microbial community data	(Segata et al, 2011)
ShotgunFunctionalizeR	A statistical test based on a Poisson model for metagenomic functional comparisons	(Kristiansson et al, 2009)
SourceTracker	A Bayesian approach to identify and quantify contaminants in a given community	(Knights et al, 2011)
		
*General toolkit*		
CAMERA	Dashboard for environmental metagenomic and genomic data, metadata, and comparative analysis tools	(Seshadri et al, 2007)
IMG/M	Integrated metagenome data management and comparative analysis system	(Markowitz et al, 2012b)
MEGAN	Software for metagenomic, metatranscriptomic, metaproteomic, and rRNA analysis	(Huson et al, 2007)
METAREP	Online storage and analysis environment for meta'omic data	(Goll et al, 2010)
MG-RAST	Storage, quality control, annotation and comparison of meta'omic samples.	(Meyer et al, 2008)
SmashCommunity	Stand-alone annotation and analysis pipeline suitable for meta'omic data	(Arumugam et al, 2010)
STAMP	Comparative meta'omics software package	(Parks and Beiko, 2010)
VAMPS	Visualization and analysis of microbial population structure	(Huse et al, 2008)

Common steps needed for metagenome and metatranscriptome interpretation include assembly, taxonomic profiling, functional profiling, ecological interaction network construction, single-cell sequencing, synthetic data simulators, and downstream statistical tests.
