# Supplementary material for: Computational meta'omics for microbial community studies
Source: Mol Syst Biol. 2013 May 14;9:666. doi: 10.1038/msb.2013.22 (PMC4039370; doi:10.1038/msb.2013.22)
Supplement: Supplementary Information — Step-by-step guide into the computational pipeline of Figure 4 [file msb201322-s1.pdf]

This tutorial is a step-by-step guide into the computational pipeline performed in Figure 4.

The tutorial is also available at:

https://bitbucket.org/nsegata/metaphlan/wiki/MetaPhlAn_Pipelines_Tutorial

This tutorial focuses on performing a comprehensive metagenomic analysis from whole-genome shotgun sequencing data. It is divided in to different steps that use the following metagenomic, computational tools: **MetaPhlAn**, **GraPhlAn**, **LEfSe**, and **HUMAnN**.

The references for these tools are reported at the end of this page. If you find these tools useful, we kindly ask you to use these references for citing. Additionally, links to the corresponding user groups are provided should you have any comments or suggestions you would like to share with us.

The tutorial below assumes you have a basic knowledge of shotgun metagenomics and of Unix-based operating systems (although you should be able to run the pipeline on MacOS and Windows, some commands may require modification). Also, Python >2.6 (but not Python 3) is required to be installed (Python 2.7 for Step 5).

Step 1: MetaPhlAn profiling of 20 HMP samples

In this step we will show how to taxonomically profile 20 metagenomic shotgun samples Human Microbiome Project (HMP). Specifically, we will look at 10 samples from the buccal mucosa and 10 from the tongue dorsum. Please note these 20 samples are not necessarily an unbiased selection of microbiomes from the over 200 buccal mucosa and tongue dorsum samples generated by the HMP.

REQUIREMENTS: BowTie2 installed and in the system path, Mercurial, basic unix commands (wget, tar).

The size of the 20 samples (60GBs) can make their download quite slow. For this reason we are also providing the overall output of Step 1 (few KBs) available for download so that one start the tutorial from Step 2 if needed.

The first operation consists in obtaining the last version of MetaPhlAn downloadable as zip, gz, or bz2 compressed archives.

In a Unix environment, you can obtain and uncompress it from the command line:

```
$ wget https://bitbucket.org/nsegata/metaphlan/get/default.tar.bz2
$ tar xjvf default.tar.bz2
$ mv *-metaphlan-* metaphlan
```

Alternatively, you can use Mercurial to obtain the package from the Bitbucket repository and keep it updated in the future:

```
$ hg clone ssh://hg@bitbucket.org/nsegata/metaphlan
```

We then navigate to the MetaPhlAn folder and create a subfolder for storing the 20 metagenomes:

```
$ cd metaphlan
$ mkdir input
```

Now, we can download the 20 samples to profile (additional information about the samples is available at the HMP DACC):

```
$ samples="SRS013506 SRS015374 SRS015646 SRS017687 SRS019221 SRS019329 SRS020336 SRS022145 SRS022532 SRS045049 SRS011243 SRS01323[illegible]
$ for s in ${samples}
$ do
$     wget http://downloads.hmpdacc.org/data/Illumina/posterior_fornix/${s}.tar.bz2 -O input/${s}.tar.bz2
$ done
```

As discussed above, this operation will likely require several hours.

Next, let's create a folder for storing the MetaPhlAn output.

```
$ mkdir profiled_samples
```

We can now profile the 10 buccal mucosa samples using MetaPhlAn. We are running MetaPhlAn using the BowTie2 engine (this step requires BowTie2 to be installed and in the system path). Notice we are piping the fastq reads directly from the compressed archive to MetaPhlAn. When piping MetaPhlAn's internal parallelization option (--nproc option) can not be used. This is available when the input is a an uncompressed file. Notice, however, that if your machine has multiple CPUs you can run multiple MetaPhlAn profiling operations in parallel.

```
$ BM_samples="SRS013506 SRS015374 SRS015646 SRS017687 SRS019221 SRS019329 SRS020336 SRS022145 SRS022532 SRS045049"
$ for s in ${BM_samples}
$ do
$     tar xjf input/${s}.tar.bz2 --to-stdout | ./metaphlan.py --bowtie2db bowtie2db/mpa --bt2_ps very-sensitive --input_type mult:
$ done
```

Please refer to the MetaPhlAn help (`$ ./metaphlan.py -h`) or to the MetaPhlAn wiki for specific information about other strategies and additional MetaPhlAn options.

Similarly, we can apply MetaPhlAn to the 10 tongue dorsum samples.

```
$ TD_samples="SRS011243 SRS013234 SRS014888 SRS015941 SRS016086 SRS016342 SRS017713 SRS019219 SRS019327 SRS043663"
$ for s in ${TD_samples}
$ do
$     tar xjf input/${s}.tar.bz2 --to-stdout | ./metaphlan.py --bowtie2db bowtie2db/mpa --bt2_ps very-sensitive --input_type mult:
$ done
```

The `profiled_samples` folder now contains 20 profiled metagenomes. Here is an example of first few lines of the `BM_SRS013506` sample output.

```
$ cat profiled_samples/BM_SRS013506.txt
$ k__Bacteria     100.0
$ k__Bacteria|p__Firmicutes       80.97874
$ k__Bacteria|p__Proteobacteria   17.17081
$ k__Bacteria|p__Proteobacteria   17.17081
$ k__Bacteria|p__Fusobacteria     0.34233
$ k__Bacteria|p__Bacteroidetes    0.17203
$ k__Bacteria|p__Firmicutes|c__Bacilli    80.62406
[truncated output]
```

The commands reported above can be retrieved as a bash script for step 1.

Step 2: MetaPhlAn output merge and visualization

In this step, we will merge the 20 profiled metagenomes in a table of relative abundances and visualize the table with a heatmap.

REQUIREMENTS: the matplotlib python library installed.

Merging the profiled metagenomes is a simple operation that can be performed with a script downloaded with MetaPhlAn and located in the `utils` folder:

```
$ mkdir output
$ utils/merge_metaphlan_tables.py profiled_samples/*.txt > output/merged_abundance_table.txt
```

The resulting table contains relative abundances with microbial clades as rows and samples as columns.

The `metaphlan_hclust_heatmap.py` script in the MetaPhlAn `plotting_scripts` folder can now be used to perform hierarchical clustering of both samples and clades to generate the heatmap:

```
$ mkdir output_images
$ plotting_scripts/metaphlan_hclust_heatmap.py -c bbcry --top 25 --minv 0.1 -s log --in output/merged_abundance_table.txt --out ou
```

Specifically, we are displaying the abundances for species only (default `--tax_lev s`), in logarithmic scale (`-s log`), reporting only the 25 most abundant clades (`--top 25`) according to the 90th percentile of the abundances in each clade (default `--perc 90`) with custom color map (`-c bbcry`). In this example, the clustering is performed with "average" linkage (default `-m average`), using "Bray-Curtis" distance for clades (default `-d braycurtis`) and "correlation" for samples (default `-f correlation`).

The resulting heatmap is shown here:

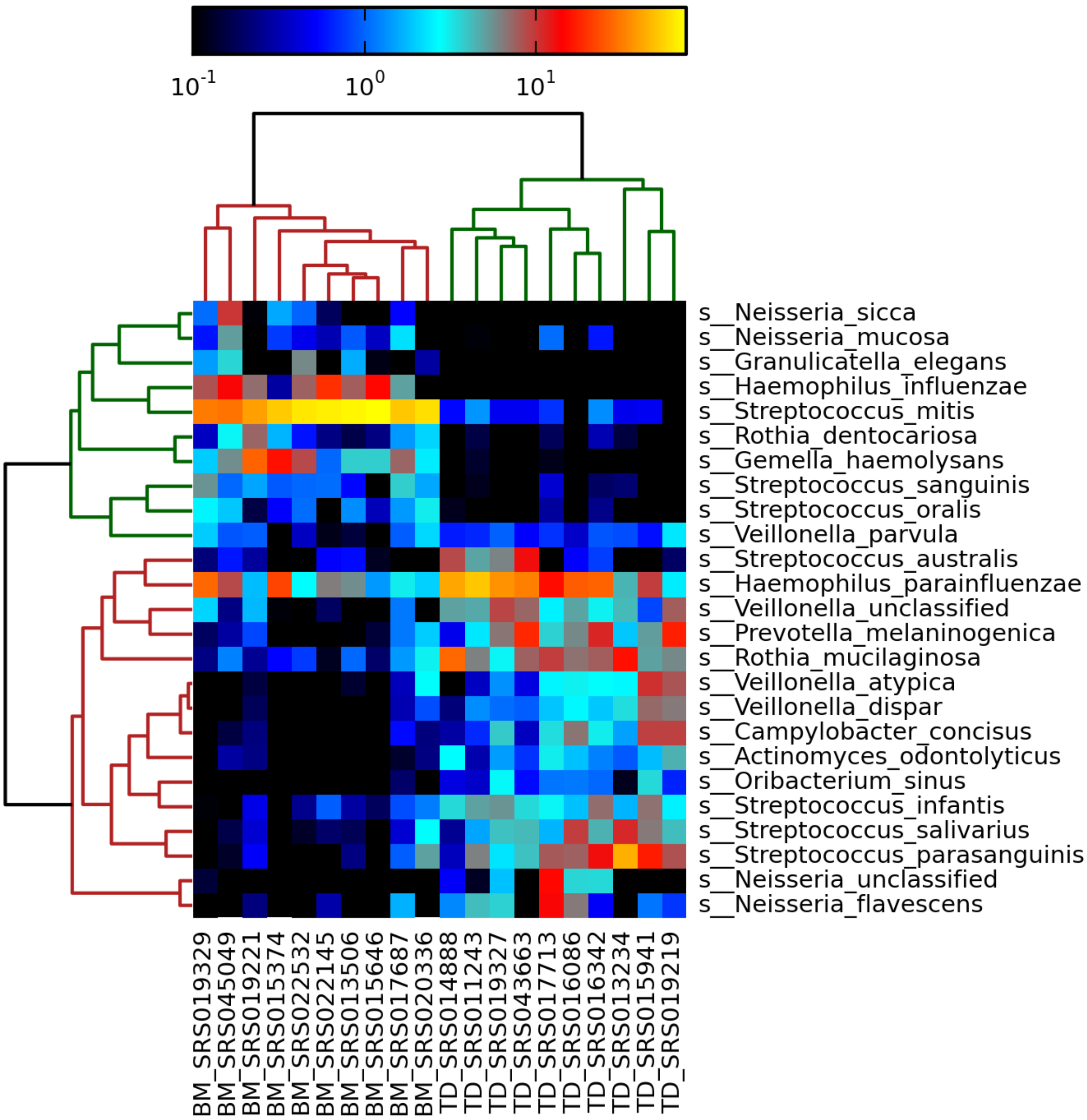

For additional customization, listed here are all available options in `metaphlan_hclust_heatmap.py` for the heatmap and/or the clustering.

```
$ plotting_scripts/metaphlan_hclust_heatmap.py -h
usage: metaphlan_hclust_heatmap.py [-h] --in INPUT_FILE --out OUTPUT_FILE
                                   [-m {single,complete,average,weighted,centroid,median,ward}]
                                   [-d {euclidean,minkowski,cityblock,seuclidean,sqeuclidean,cosine,correlation,hamming,jaccard,ch
                                   [-f {euclidean,minkowski,cityblock,seuclidean,sqeuclidean,cosine,correlation,hamming,jaccard,ch
                                   [-s scale norm] [-x X] [-y Y] [--minv MINV]
                                   [--maxv max value]
                                   [--tax_lev TAXONOMIC_LEVEL] [--perc PERC]
                                   [--top TOP] [--sdend_h SDEND_H]
                                   [--fdend_w FDEND_W] [--cm_h CM_H]
                                   [--cm_ticks label for ticks of the colormap]
                                   [--font_size FONT_SIZE]
                                   [--clust_line_w CLUST_LINE_W]
                                   [-c {Accent,Blues,BrBG,BuGn,BuPu,Dark2,GnBu,Greens,Greys,OrRd,Oranges,PRGn,Paired,Pastel1,Paste

This scripts generates heatmaps with hierarchical clustering of both samples
and microbial clades. The script can also subsample the number of clades to
display based on the their nth percentile abundance value in each sample

optional arguments:
```

```
  -h, --help            show this help message and exit
  --in INPUT_FILE       The input file of microbial relative abundances. This
                        file is typically obtained with the
                        "utils/merge_metaphlan_tables.py"
  --out OUTPUT_FILE     The output image. The extension of the file determines
                        the image format. png, pdf, and svg are the preferred
                        format
  -m {single,complete,average,weighted,centroid,median,ward}
                        The hierarchical clustering method, default is
                        "average"
  -d {euclidean,minkowski,cityblock,seuclidean,sqeuclidean,cosine,correlation,hamming,jaccard,chebyshev,canberra,braycurtis,mahala
                        The distance function for samples. Default is
                        "braycurtis"
  -f {euclidean,minkowski,cityblock,seuclidean,sqeuclidean,cosine,correlation,hamming,jaccard,chebyshev,canberra,braycurtis,mahala
                        The distance function for microbes. Default is
                        "correlation"
  -s scale norm
  -x X                  Width of heatmap cells. Automatically set, this option
                        should not be necessary unless for very large heatmaps
  -y Y                  Height of heatmap cells. Automatically set, this
                        option should not be necessary unless for very large
                        heatmaps
  --minv MINV           Minimum value to display. Default is 0.0, values
                        around 0.001 are also reasonable
  --maxv max value      Maximum value to display. Default is maximum value
                        present, can be set e.g. to 100 to display the full
                        scale
  --tax_lev TAXONOMIC_LEVEL
                        The taxonomic level to display: 'a' : all taxonomic
                        levels 'k' : kingdoms (Bacteria and Archaea) only 'p'
                        : phyla only 'c' : classes only 'o' : orders only 'f'
                        : families only 'g' : genera only 's' : species only
                        [default 's']
  --perc PERC           Percentile to be used for ordering the microbes in
                        order to select with --top the most abundant microbes
                        only. Default is 90
  --top TOP             Display the --top most abundant microbes only
                        (ordering based on --perc)
  --sdend_h SDEND_H     Set the height of the sample dendrogram. Default is
                        0.1
  --fdend_w FDEND_W     Set the width of the microbes dendrogram. Default is
                        0.1
  --cm_h CM_H           Set the height of the colormap. Default = 0.03
  --cm_ticks label for ticks of the colormap
  --font_size FONT_SIZE
                        Set label font sizes. Default is 7
  --clust_line_w CLUST_LINE_W
                        Set the line width for the dendrograms
  -c {Accent,Blues,BrBG,BuGn,BuPu,Dark2,GnBu,Greens,Greys,OrRd,Oranges,PRGn,Paired,Pastel1,Pastel2,PiYG,PuBu,PuBuGn,PuOr,PuRd,Purp
                        Set the colormap. Default is "jet".
```

The commands reported above can be retrieved as a bash script for step 2.

Step 3: GraPhlAn visualization of single and multiple samples

In this step we describe some approaches to graphically represent single profiled samples or a merged table of relative abundances.

REQUIREMENTS: GraPhlAn installed (and in the system path), and the matplotlib python library. GraPhlAn can be downloaded using Mercurial: `hg clone ssh://hg@bitbucket.org/nsegata/graphlan`.

The `metaphlan2graphlan.py` script in the plotting_scripts folder can generate the two required input files for GraPhlAn which are (i) a tree structure to represent and (ii) graphical annotation options for the tree.

```
$ mkdir tmp
$ plotting_scripts/metaphlan2graphlan.py profiled_samples/BM_SRS013506.txt  --tree_file tmp/BM_SRS013506.tree.txt --annot_file tmp
```

With these two generated files we can now run GraPhlAn (please refer to GraPhlAn project page for detailed information and additional customization options).

```
$ graphlan_annotate.py --annot tmp/BM_SRS013506.annot.txt tmp/BM_SRS013506.tree.txt tmp/BM_SRS013506.xml
$ graphlan.py --dpi 200 tmp/BM_SRS013506.xml output_images/BM_SRS013506.png
```

Here is the resulting image:

A:g__Gemella
B:g__Streptococcus
C:g__Haemophilus

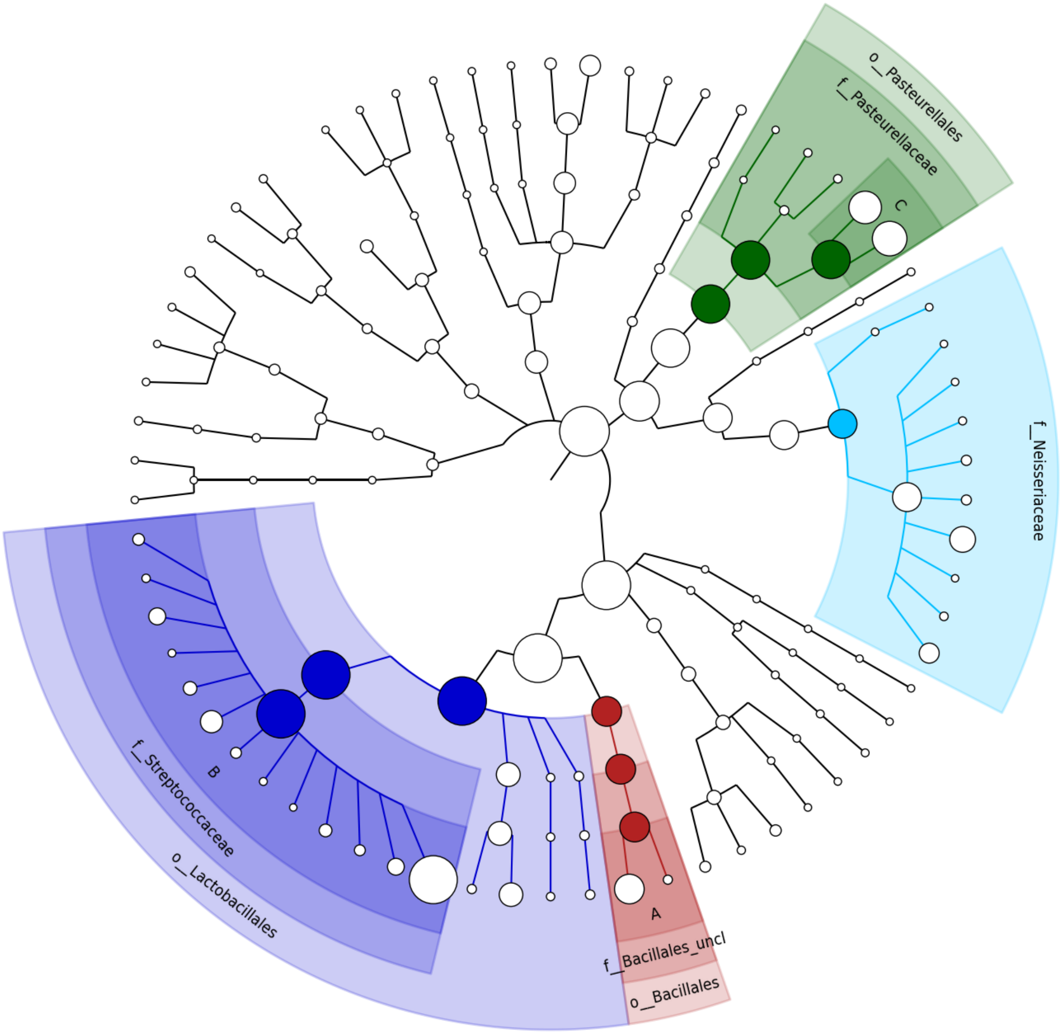

Images for all samples can be created with a script similar to this example:

```
$ mkdir -p tmp
$ for file in profiled_samples/*
$ do
$     filename=`basename ${file}`
$     samplename=${filename%\.*}
$     plotting_scripts/metaphlan2graphlan.py ${file}  --tree_file tmp/${samplename}.tree.txt --annot_file tmp/${samplename}.annot
$     graphlan_annotate.py --annot tmp/${samplename}.annot.txt tmp/${samplename}.tree.txt tmp/${samplename}.xml
$     graphlan.py --dpi 200 tmp/${samplename}.xml output_images/${samplename}.png
$ done
```

There are additional options for customization that can be used to modify the output circular tree. Specifically, several options can be set in the `metaphlan2graphlan.py` script to control the number of annotated clades shown (`--max_annot_clades`, default 10), to set the starting and ending annotated taxonomic levels (`--min_annot_lev` and `--max_annot_lev`), and many more options. All current options are listed below:

```
$ plotting_scripts/metaphlan2graphlan.py -h
usage: metaphlan2graphlan.py [-h] --tree_file TREE_FILE --annot_file
                             ANNOT_FILE [--max_annot_clades MAX_ANNOT_CLADES]
                             [--min_annot_lev MIN_ANNOT_LEV]
                             [--max_annot_lev MAX_ANNOT_LEV]
```

```
                             [--ext_keys_start_lev EXT_KEYS_START_LEV]
                             [--coloring_lev COLORING_LEV]
                             [INPUT_FILE]

DESCRIPTION
  metahplan2graphlan.py version 0.9 (17th March 2013)
  Convert MetaPhlAn outputs to GraPhlAn imput format.
  AUTHORS: Nicola Segata (nicola.segata@unitn.it)

EXAMPLE
  metaphlan2graphlan.py metahplan-out.txt --tree_file out_graphlan_tree.txt --annot_file out_annotation_options.txt

positional arguments:
  INPUT_FILE            Merged MetaPhlAn data.

optional arguments:
  -h, --help            show this help message and exit
  --tree_file TREE_FILE
                        The output tree file for GraPhlAn
  --annot_file ANNOT_FILE
                        The annotation file for GraPhlAn
  --max_annot_clades MAX_ANNOT_CLADES
                        The maximum number of clades to annotate [default 10]
  --min_annot_lev MIN_ANNOT_LEV
                        The minimum number of levels required to label a clade [default 2, meaning microbial orders]
  --max_annot_lev MAX_ANNOT_LEV
                        The maximum number of levels required to label a clade [default 5, meaning microbial species]
  --ext_keys_start_lev EXT_KEYS_START_LEV
                        The level at which annotations are added using external legend keys [default 5, meaning microbial species]
  --coloring_lev COLORING_LEV
                        The level used for color differentiation [default 3, meaning microbial families]
```

The annotation file can be edited manually and/or with ad-hoc scripts for a higher degree of customization. Please refer to the GraPhlAn readme file and to these examples for additional information.

The same script can also be applied to the relative abundance table of 20 merged samples. In this case, the abundances reported refer to the overall average of each clade across all the samples in the table.

```
$ plotting_scripts/metaphlan2graphlan.py output/merged_abundance_table.txt  --tree_file tmp/merged.tree.txt --annot_file tmp/merge
$ graphlan_annotate.py --annot tmp/merged.annot.txt tmp/merged.tree.txt tmp/merged.xml
$ graphlan.py --dpi 200 tmp/merged.xml output_images/merged.png
```

As an example of manually and script-based modification of the "annotation" file provided in this file, we can produce comparisons between classes. Note, this script also includes information about biomarkers (detailed in Step 4: Taxonomic Biomarker discovery with LEfSe). Step 4. The resulting GraPhlAn image is reported below:

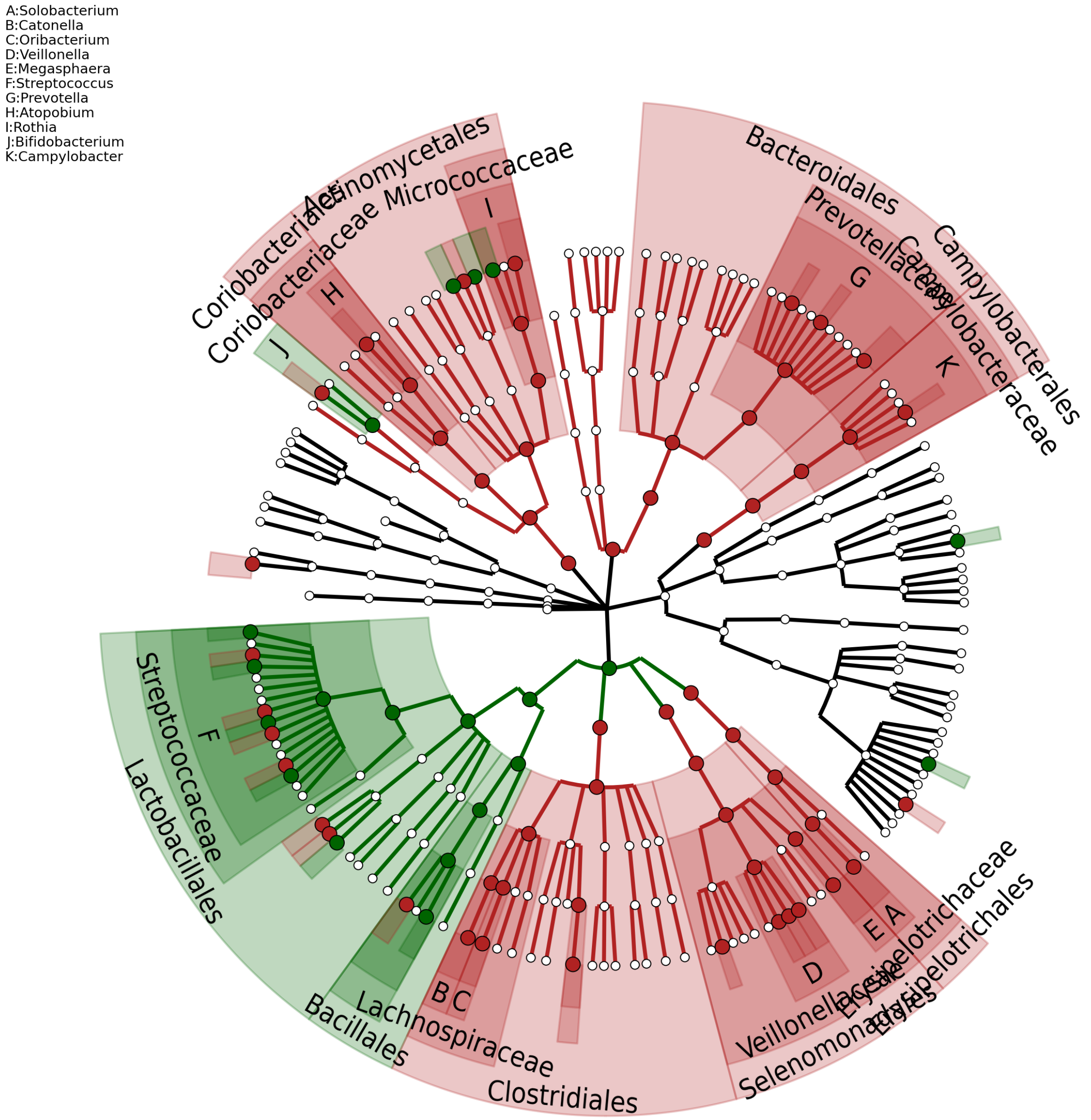

Notice also that MetaPhlAn output can be exported to Krona (another popular visualization tool) using the script `metaphlan2krona.py` in the `conversion_scripts` folder downloaded as part of the MetaPhlAn package.

The commands reported above can be retrieved as a bash script for step 3.

Step 4: Taxonomic biomarker discovery with LEfSe

In this step we show how to perform the taxonomic biomarker discovery operation using LEfSe.

REQUIREMENTS: LEfSe installed (and in the system path), and the matplotlib python library installed. Alternatively, the users are welcome to use the Galaxy interface for LEfSe. LEfSe can be downloaded using using Mercurial: `hg clone ssh://hg@bitbucket.org/nsegata/lefse` or using the direct links to the zip, gz, or bz2 archives. Notice that LEfSe has some additional requirements

We first need to specify the conditions (or classes) used for the biomarker discovery. Examples could be host disease states from which a gut microbiome was sampled, or environmental conditions (e.g. pH, environmental contaminant) from which a microbial community was sampled. In this tutorial, we will use body sites (contrasting the tongue dorsum microbiome with the buccal mucosa microbiome). Before beginning, we need to convert the sample names into consistent class names. This can easily be done by manually editing the `output/merged_abundance_table.txt` generated in the steps above or using the following "sed" based Unix command:

```
$ sed 's/\([A-Z][A-Z]\)_\w*/\1/g' output/merged_abundance_table.txt > tmp/merged_abundance_table.4lefse.txt
```

The first LEfSe step consists of formatting the input table, making sure the class information is in the first row and scaling the values in [0,1M] which is useful for numerical computational reasons.

```
$ format_input.py tmp/merged_abundance_table.4lefse.txt tmp/merged_abundance_table.lefse -c 1 -o 1000000
```

Now, the LEfSe biomarker discovery tool can be used with default statistical options. Here we change one default parameter to increase the threshold on the LDA effect size from 2 (default) to 4.

```
$ run_lefse.py tmp/merged_abundance_table.lefse tmp/merged_abundance_table.lefse.out -l 4
```

The results of the operation can now be displayed plotting the resulting list of biomarkers with corresponding effect sizes.

```
$ plot_res.py --dpi 300 tmp/merged_abundance_table.lefse.out output_images/lefse_biomarkers.png
```

The resulting image (`output_images/lefse_biomarkers.png`) is shown below. (For this specific image we increased the LDA threshold to 4.7 in order to display a more compact image with fewer biomarkers).

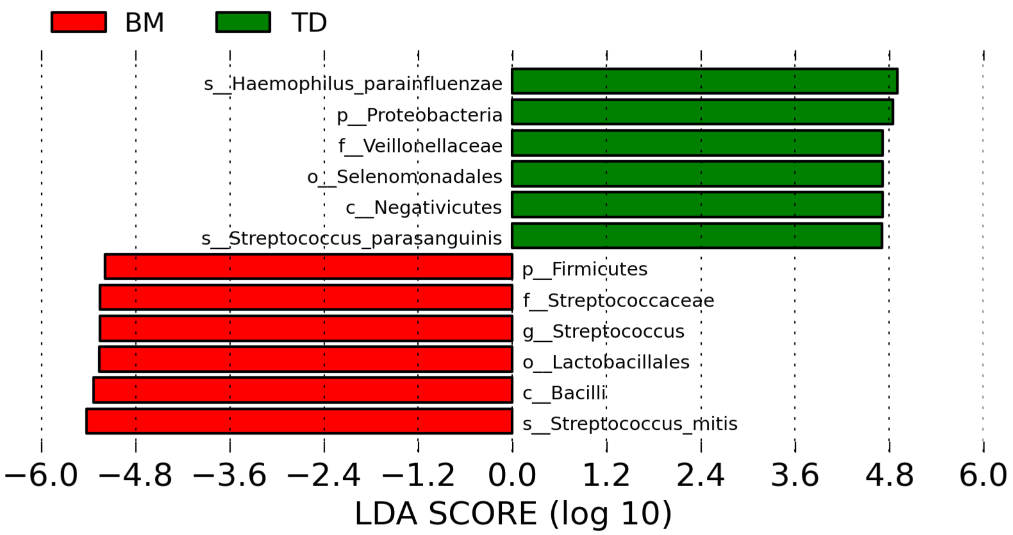

Another complementary visualization focuses on showing the biomarkers on the underlying taxonomic tree.

```
$ plot_cladogram.py --dpi 300 --format png tmp/merged_abundance_table.lefse.out output_images/lefse_biomarkers_cladogram.png
```

The default plotting output is shown here.

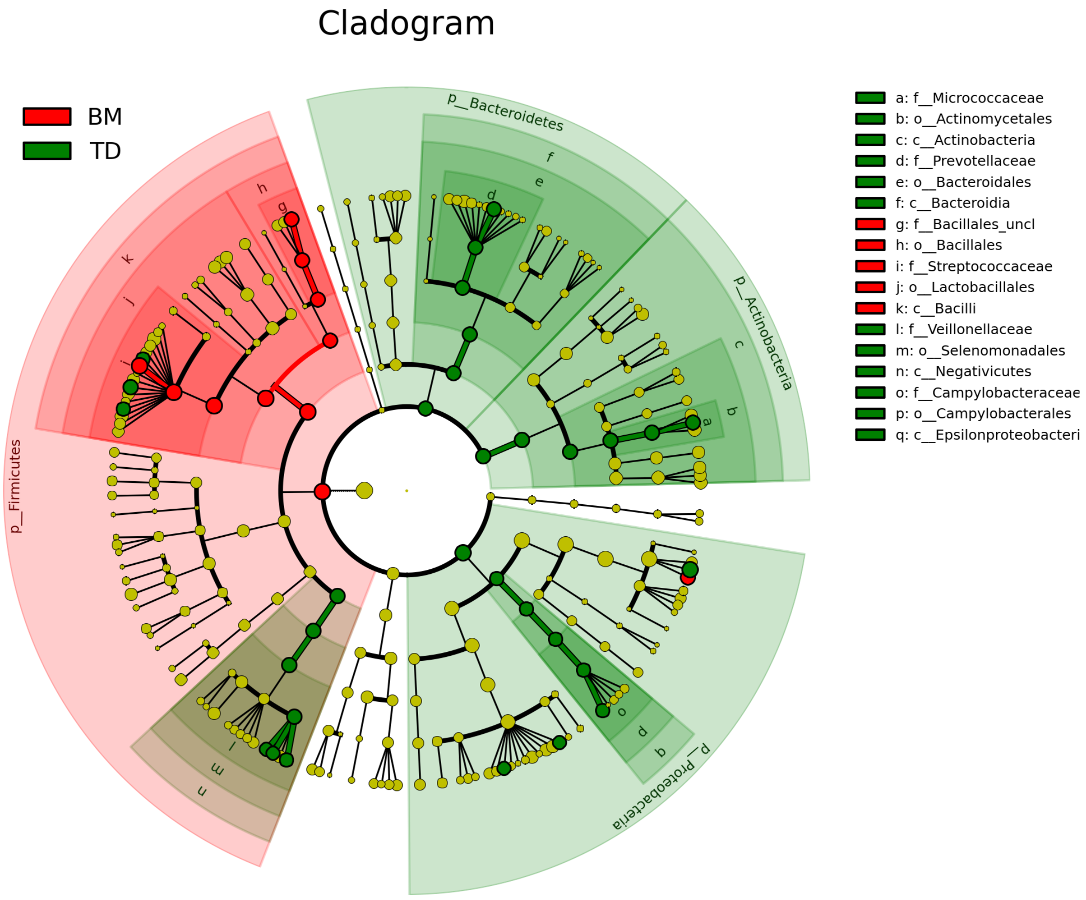

Notice that the cladograms produced by LEfSe can be less graphically appealing and detailed than those built using GraPhlAn. Using the `tmp/merged_abundance_table.lefse.out` file and, a combination of scripting or manual editing, it is possible to obtain an improved graphical output for LEfSe similar to the cladogram reported at the end of Step 3 (which is reporting the same biomarkers as the cladogram above).

The commands reported above can be retrieved as a bash script for step 4.

Step 5: Metabolic profiling with HUMAnN

In this step, we perform a metabolic profiling of metagenomic datasets using HUMAnN applied to the 20 samples taxonomically profiled above. Once the metabolic profile has been generated, the above steps 2-4 can be performed.

REQUIREMENTS: HUMAnN, scons, the KEGG protein DB. HUMAnN can be obtained using Mercurial: `hg clone ssh://hg@bitbucket.org/chuttenh/humann` or using the direct links to the zip, gz, or bz2 archives.

Metabolic profiles of the 20 HMP samples with HUMAnN can be generated with the following steps:

- Perform a translated search (using blastx or usearch) against the KEGG DB. Since HUMAnN's development KEGG has become commercial, we are currently developing support for other data sources.
- Place the translated BLAST results using KEGG gene identifiers in the `input` directory (optionally can be gzipped or bzipped, several other formats can be enabled editing the settings in the `SConstruct` file).
- Run the `scons` command, optionally parallelizing multiple analyses using the `-j` flag. Results will be placed in the "output" directory.

Similar to Step 1, we provide the HUMAnN output for users who want to perform the downstream analysis pipeline but avoid the computational intensive steps above. The 20 samples profiled with HUMAnN are available here.

Using HUMAnN output, you can perform the metabolic counterpart of the taxonomic pipeline presented in this tutorial. With a table of metabolomic abundances, the previous steps 2-4 can be performed with little to no modification. We report below the first command to obtain the merged table of metabolic abundances.

```
$ mkdir output
$ utils/merge_metaphlan_tables.py humann_profiling/*.txt > output/merged_humann_abundance_table.txt
```

Citation and additional information and support

MetaPhlAn

"Metagenomic microbial community profiling using unique clade-specific marker genes"
Nicola Segata, Levi Waldron, Annalisa Ballarini, Vagheesh Narasimhan, Olivier Jousson, Curtis Huttenhower.
Nature Methods, 8, 811–814, 2012

Source code: https://bitbucket.org/nsegata/metaphlan

Galaxy interface: http://huttenhower.sph.harvard.edu/galaxy/root?tool_id=metaphlan

User Group: https://groups.google.com/forum/?fromgroups#!forum/metaphlan-users

GraPhlAn

"Graphical Phylogenetic Analysis for Metagenomic studies"
In preparation

Source code: https://bitbucket.org/nsegata/graphlan

Galaxy interface: http://huttenhower.sph.harvard.edu/galaxy/root?tool_id=graphlan

User Group: https://groups.google.com/forum/?fromgroups=#!forum/graphlan-users

LEfSe

"Metagenomic biomarker discovery and explanation"
Nicola Segata, Jacques Izard, Levi Waldron, Dirk Gevers, Larisa Miropolsky, Wendy S Garrett, and Curtis Huttenhower
Genome Biology, 12:R60, 2011

Source code: https://bitbucket.org/nsegata/lefse

Galaxy interface: http://huttenhower.sph.harvard.edu/galaxy/root?tool_id=lefse_upload

HUMAnN

"Metabolic Reconstruction for Metagenomic Data and Its Application to the Human Microbiome"
Sahar Abubucker, Nicola Segata, Johannes Goll, Alyxandria M. Schubert, Jacques Izard, Brandi L. Cantarel, Beltran Rodriguez-Mueller, Jeremy Zucker, Mathangi Thiagarajan, Bernard Henrissat, Owen White, Scott T. Kelley, Barbara Methé, Patrick D. Schloss, Dirk Gevers, Makedonka Mitreva, Curtis Huttenhower
PLoS Computational Biology 8(6), 2012

Source code: https://bitbucket.org/chuttenh/humann

User Group: https://groups.google.com/forum/?fromgroups=#!forum/humann-users
